# Filamentous fungi-like secretory pathway strayed in a yeast system: peculiarities of *Yarrowia lipolytica* secretory pathway underlying its extraordinary performance

**DOI:** 10.1007/s00253-018-9450-2

**Published:** 2018-10-23

**Authors:** Ewelina Celińska, Jean-Marc Nicaud

**Affiliations:** 1 Department of Biotechnology and Food Microbiology, Poznan University of Life Sciences, ul. Wojska Polskiego 48, 60-627 Poznań, Poland; 2 INRA-AgroParisTech, UMR1319, Team BIMLip: Integrative Metabolism of Microbial Lipids, Micalis Institute, Domaine de Vilvert, 78352 Jouy-en-Josas, France

**Keywords:** *Yarrowia lipolytica*, Protein secretion, Protein folding and maturation, Nonconventional expression system, Secretory pathway, Heterologous protein

## Abstract

Microbial production of secretory proteins constitutes one of the key branches of current industrial biotechnology, earning billion dollar (USD) revenues each year. That industrial branch strongly relies on fluent operation of the secretory machinery within a microbial cell. The secretory machinery, directing the nascent polypeptide to its final destination, constitutes a highly complex system located across the eukaryotic cell. Numerous molecular identities of diverse structure and function not only build the advanced network assisting folding, maturation and secretion of polypeptides but also serve as sensors and effectors of quality control points. All these events must be harmoniously orchestrated to enable fluent processing of the protein traffic. Availability of these elements is considered to be the limiting factor determining capacity of protein traffic, which is of crucial importance upon biotechnological production of secretory proteins. The main purpose of this work is to review and discuss findings concerning secretory machinery operating in a non-conventional yeast species, *Yarrowia lipolytica*, and to highlight peculiarities of this system prompting its use as the production host. The reviewed literature supports the thesis that secretory machinery in *Y. lipolytica* is characterized by significantly higher complexity than a canonical yeast protein secretion pathway, making it more similar to filamentous fungi-like systems in this regard.

## Introduction

Proteins, like bulk industrial enzymes, enzymatic preparations used in research and foods processing, or polypeptides applied in diagnostics and therapy, are currently mainly produced by native or genetically engineered microorganisms. Due to technical limitations, it seems that completely synthetic proteins generated in a course of a chemical, and not biochemical process, are still far from reality, unlike for example synthetic chromosomes (Annaluru et al. 2014). Initially, proteins applied in various fields of human activity, like cheese-making or diabetes treatment, were isolated from their natural origin—calf stomach or porcine pancreas. However, such methods impose significant problems, like limited availability of the source material, or risk of pathogen transfer. Hence, acquisition of a given protein counterpart from safe microorganisms, or production of an “original” polypeptide in an engineered microbial host constitutes a superior and highly attractive alternative. Depending on the microbial host and characteristics of the targeted polypeptide, the protein product may be secreted outside the cell or remain retained inside its structures. The former strategy is highly desired as it greatly simplifies assaying the protein product, its isolation and purification. Moreover, for acquisition of the final characteristics, many proteins require a complete maturation process, and hence they need to traverse across the whole secretory pathway. For all these reasons, microbial production of secretory proteins constitutes one of the key branches of current industrial biotechnology, earning billion dollar (USD) revenues each year (Graf et al. 2009; Çelik and Çalık 2011; Liu et al. 2012; Delic et al. 2013). Noteworthy, this industrial branch strongly relies on fluent operation of the secretory machinery within a eukaryotic cell.

**Fig. 1:**
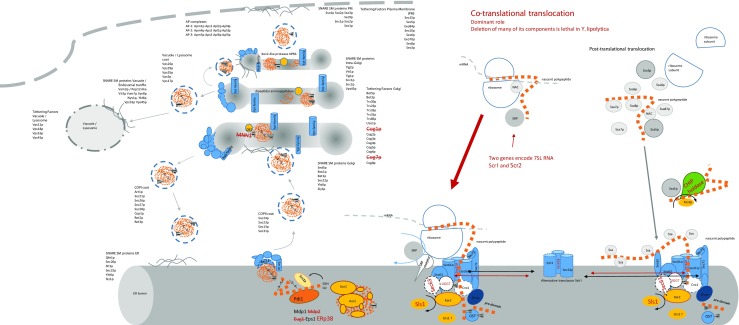
Secretory pathway with known specificities present in *Yarrowia lipolytica*. Detailed information about the role of individual elements of secretory machinery depicted in the figure can be found in the text. All molecular identities that are depicted in the figure were identified in *Y. lipolytica* by either proteomics or comparative genomics approaches (Swennen and Beckerich 2007; Swennen et al. 2010; Delic et al. 2013). Any specific traits that differentiate *Y. lipolytica* from the model yeast are depicted in dark red (crossing outs—lack of a gene encoding a given activity; large font—unique presence of a gene encoding a given activity in *Y. lipolytica*, or its dominant role, e.g., Sls1 NEF). Unknown function of Lhs1 GEF was indicated by quotation mark. The dominant role of the co-translational translocation pathway is indicated by thicker, red arrow, compared to thin gray marking post-translational translocation. Nascent polypeptide is marked in orange. Lined triangles—N-glycans. Coating proteins, tethering factors, SNARE, and SM proteins were listed as identified by proteomics analysis in Swennen and Beckerich (2007)

**Fig. 2:**
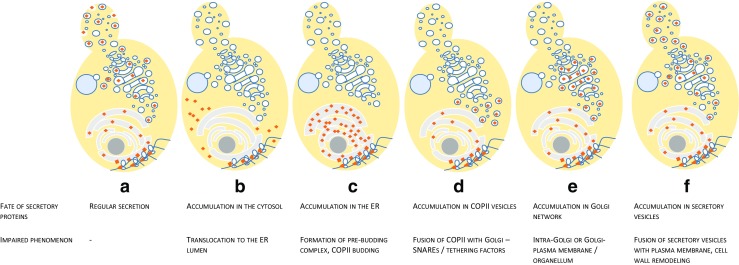
Simplified scheme representing possible bottlenecks of the secretory pathway. Dark gray nucleus, light gray endoplasmic reticulum, white Golgi apparatus and membrane transport vesicles (all types—COPI, COPII, secretory), light blue organellum (vacuole, lysosome), and orange squares polypeptides; ribosomes attached to the ER on the bottom of the schemes. Exemplary dysfunctions triggering depicted phenomena can be found in the main text

The secretory pathway directing the nascent polypeptides to the extracellular space, cell wall, plasma membrane, or organelles constitutes a highly complex system, spanning different compartments of a eukaryotic cell (Fig. 1). Numerous molecular identities of diverse structure and function build the advanced network assisting folding, maturation, and secretion of polypeptides. Large proportion of the proteins involved in the secretory pathway serve as sensors and effectors of quality control points. All these phenomena must be harmoniously orchestrated to enable fluent processing of protein traffic. Many of the molecular events taking place during protein maturation and secretion have stochastic character and thus not only require considerable amounts of energy, building elements and cofactors, but also sequester specialized proteins and membranes. Availability of these elements is considered to be the limiting factor determining capacity of the secretory pathway (Xu and Robinson 2009; Tyo et al. 2012). Excessive production of a given secretory protein inevitably leads to overloading the secretion pathway, accumulation of unfolded protein, and physiological stress (Mattanovich et al. 2004; Matsumoto et al. 2005; Tyo et al. 2012; Hou et al. 2014). As evidenced, unbalanced overexpression may also trigger instability of the producer cells (Ogrydziak and Nicaud 2012). The necessity to carefully adjust the protein production levels to the host cell capacities was recently exemplified by Dulermo et al. (2017). It was demonstrated that excessive overproduction of heterologous enzymatic proteins, while leading to higher secretion of the polypeptides, caused a decrease in the overall specific activity of the secreted enzymes. The authors concluded that excessive protein production could negatively affect protein folding due to deficiency in chaperones and saturation of the secretion machinery (Dulermo et al. 2017). It has been postulated that folding, maturation, and intracellular transportation constitute the main bottlenecks of the overall process of recombinant protein production (Gasser et al. 2007a; de Ruijter and Frey 2015; Puxbaum et al. 2015; Tang et al. 2015). Depending on the specific properties of the targeted polypeptide, i.e., primary and secondary structure, oligomerization, glycosylation, and the ultimate destination, different phenomena may limit the overall efficiency of the secretory protein synthesis. Correspondingly, specific characteristics of the host cell may impose bottlenecks or promote enhanced secretory capacity at different stages of the protein formation. Consequently, the protein of interest may accumulate at different levels of the polypeptide transit, depending on the bottleneck’s character (Fig. 2).

Extensive reviews covering research and progress in the field of heterologous protein production in a non-conventional yeast species *Yarrowia lipolytica* have been already published (Nicaud et al. 2002; Madzak et al. 2004; Madzak and Beckerich 2013; Madzak 2015), even very recently (Madzak 2018). For summary on elucidation of useful genetic elements and development of genetic engineering tools applied in the secretory protein production, as well as overview on *Y. lipolytica*’s fields of exploitation, the reader should refer to those articles. Current state of knowledge on the secretory pathways in several different yeast species based on genomic sequence data mining has been recently comprehensively reviewed (Delic et al. 2013). In the present work, we review and discuss findings concerning the secretory machinery operating in *Y. lipolytica*. Moreover, we highlight specific qualities of *Y. lipolytica* secretory pathway and indicate their practical consequences for heterologous protein production, as heterologous protein destined for secretion follows the same secretion route as native polypeptides (Marsalek et al. 2017). The major purpose of this review was to pinpoint the value of *Y. lipolytica* as a heterologous protein producer from a perspective of its biology.

## General overview of the canonical protein secretion pathway in yeast cells

An overview of secretory machineries operating in different yeast species presented in Delic et al. (2013) demonstrated that in spite of huge genetic and physiological diversity of yeast species, some core elements of the secretory pathway frequently remain conserved. In this work, *S. cerevisiae* was used as a benchmark, since most comprehensive experimental evidence on biology of the secretory pathway has been accumulated for this species. In yeast, as in higher eukaryotes, synthesis of secretory proteins (including plasma membrane- and organelles-associated polypeptides) may be initiated on free cytoplasmic ribosomes or those already associated with a membrane of ER or a target organelle, through an intrinsic affinity for translocon complex (George et al. 2002). Secretory proteins are distinguished from cytosolic polypeptides based on the structure of their N-terminal element protruding from the ribosomes. Immediately after the initial N-terminal domain of the nascent polypeptide is extruded from the ribosome, it is first recognized by NAC (nascent-polypeptide-associated complex) which is a peripheral component of cytoplasmic ribosomes (George et al. 2002). The role of NAC is to provide a protective environment for short, amino-terminal nascent chains, either targeted for secretion or not. The nascent polypeptides can be directed to the secretory pathway either co- or post-translationally, depending on the character of the N-terminal element as well as innate preference of the host cell (Fig. 1). In the post-translational translocation pathway (also called SRP-independent; SRP – signal recognition particle), the polypeptide is translocated through the ER membrane after dissociation of the ribosome followed by specific recognition of protected polypeptide by the components of the translocation apparatus (Brodsky and Schekman 1993). The protection of the released polypeptide in extended conformation (translocation competent state) is secured by the action of cytosolic chaperones (Hsc70s; heat-shock-cognate 70-kDa class; Ssas). As discussed in Delic et al. (2013), post-translational translocation is considered less robust than the alternative route. The second translocation mechanisms (SRP-dependent) require SRP and occurs simultaneously with the translation process. As mentioned above, immediately after the initial N-terminal domain of the nascent polypeptide is extruded from the ribosome, it is first recognized by NAC. It has been demonstrated that depletion of NAC from ribosomes carrying nascent polypeptides allows the SRP to crosslink to polypeptides irrespective of whether or not they contain signal peptides, leading to mistranslocation into the ER (Wiedmann et al. 1994). In this sense, NAC enables specific binding of SRP with signal peptides and not any nascent polypeptide. SRP is a soluble IIS ribonucleoprotein complex composed of a 7SL RNA (300 nt long) and six to seven polypeptides, which functions as an adapter between the translational machinery in the cytoplasm and the translocational machinery in the ER membrane (Blobel et al. 1979; Hortsch et al. 1986; Brodsky 1998). Binding of SRP to the nascent polypeptide leads to a pause in the protein elongation, and the whole ribosome-polypeptide-SRP complex is targeted to an SRP receptor located on the surface of ER membrane, where the ribosome interacts with the translocation channel elements by tight junction (Sec61p complex). This interaction results in release of SRP, further arrest in the protein elongation, and finally translocation of the N-terminal polypeptide into the ER lumen. The translocation pore in the yeast cells is a multicomponent complex that forms an aqueous pore through the ER membrane. Indeed, during the traverse through the whole secretory pathway, the ER membrane crossing is the sole membrane-crossing event that the polypeptide is subjected to. Sec61 and Ssh1 are the two translocon pore complexes known to operate in *S. cerevisiae* (Delic et al. 2013). Both translocons have partially overlapping scope of client polypeptides, but Sec61 is known to be less stringent in selection and accepts a wide spectrum signal peptides. The translocon Sec61 is composed of heterotrimers Sec61αβγ (Sec61p, Sbh1p, Sss1p in *S. cerevisiae*), the heteromer Sec62p/Sec63p, and some other specific proteins like Sec71p/Sec72p, which all oligomerize to form the channel (Feldheim et al. 1992). Sss1p is the sole shared element between the two translocons in *S. cerevisiae* (Delic et al. 2013). On the lumenal side of the ER membrane, a key multifunctional Hsp70 chaperone—Kar2 and its Hsp40 cochaperone (Sec63p, bearing J domain)—“pulls” the polypeptide inside this compartment (Panzner et al. 1995). Sec63p is known to play several different roles upon polypeptide translocation: (i) binding Sec62p subunit, which is exclusive for post-translational translocon, (ii) gating the translocation pore in cooperation with Kar2, and (iii) stabilizing the pores in both co- and post-translational translocation pathways. The multifunctional Kar2 chaperone binds to exposed hydrophobic domains of unfolded polypetides and acts as molecular ratchet during translocation (Matlack et al. 1999). Kar2 is known to bear ATPase activity, which is modulated by the action of NEF (nucleotide exchange factor), facilitating exchange of ADP with ATP. Two NEFs are known in *S. cerevisiae*: Lhs1p and Sil1p, with the former having a dominant role in this yeast species (Steel et al. 2004). Upon translocation of the N-terminal domain of the polypeptide, its cleavage by a specific signal peptidase (SPase) and core glycosylation by oligosaccharyl transferase (OST) is executed by the ER resident proteins. The translocated polypeptide is then subjected to a series of manipulations in the ER, leading to acquisition of correct folding and mature structure, including disulfide bonds formation. Proteolytic cleavage by specific proteases or glycosylation by specific glycosidases is mainly executed in the Golgi compartment. The process of disulfide bond formation is mediated by Pdi1p and Ero1p (protein disulfide isomerase and Pdi1p oxidase, respectively) and relies on stochastic oxidation-reduction of cysteine side chains, which consumes considerable amounts of oxidating and reducing agents (O2 and GSH, respectively). If unbalanced, this process may lead to excessive formation of reactive oxygen species and heavy oxidative stress, and hence is considered to be particularly sensitive upon heterologous protein overexpression, even those not bearing any disulfide bridges in their secondary structures (Hou et al. 2012b; Tyo et al. 2012; Guerrero-Gómez et al. 2018). As discussed in Guerrero-Gómez et al. (2018), neither thioredoxin nor glutathione redox systems representatives were found in the ER, and the identity of the enzymatic systems providing reducing equivalents to ER-resident PDIs remained elusive for long time. The current model implies that cytoplasmic thioredoxin shuttles electrons into the ER to reduce oxidized PDIs (Poet et al. 2017), while GSH is actively transported from cytoplasm by specific transporters (Ponsero et al. 2017). Along the protein traverse across the secretory pathway, it is subjected to further specific modifications, multiple quality-control points, and sorting to finally reach the destination site. Upon exit from the ER, the secretory polypeptides undergo sorting events. If properly folded, they are directly or indirectly recognized by the coat proteins of budding vesicles for anterograde transport, while unfolded or misfolded proteins are retained in the ER by a quality control mechanism. The quality-control systems disable incorrectly folded or modified proteins to continue their route through the secretory pathway. The polypeptides which could not be correctly processed are degraded by the cytoplasmic ubiquitin-proteasome system via ER-associated degradation (ERAD), which protects the cell from the UPR-related stress or cellular dysfunction (Stein et al. 2014; Preston et al. 2018; Berner et al. 2018). Correctly folded proteins traverse from ER to Golgi compartment and to any further destination via vesicular transportation (McNew et al. 1998; Gasser et al. 2007b; Gasser et al. 2007a; Johansen et al. 2012; Hou et al. 2012a; Puxbaum et al. 2016; Marsalek et al. 2017). Correct sorting and targeting of the polypeptides require concerted action of an extensive panel of molecular identities, i.e., vesicle coat proteins, tethering factors, membrane recycling factors, Ypt/Rab GTPases, SNARE complexes (soluble NSF(N-ethylmaleimide-sensitive factor) attachment protein receptor), expansins, and exocytosis promoting kinases (Gasser et al. 2007b; Swennen and Beckerich 2007; Idiris et al. 2010a; Liu et al. 2014; Puxbaum et al. 2015). This extensive net of vesicle transportation and sorting is initiated by promoting ER membrane curvature by the action of Sar1p GTPase by guanine exchange factor Sec12p, being ER-specific membrane-bound protein (Nakano and Muramatsu 1989; D’Enfert et al. 1991). Activated Sar1p-GTP recruits the heterodimer Sec23p-Sec24p and Sec13p-Sec31p (COPII coating proteins), stabilizing the whole pre-budding complex (dysfunction in this process may result in situation presented in Fig. 2c). Retrograde vesicle transportation from cis-Golgi to ER and between Golgi compartments is assisted by COPI coatomer, consisting of large protein subcomplexes, including Sec21p, Sec26p/27p/28p, Ret2/3p, Cop1p, and Arf1p. Finally, ER-to-Golgi, intra-Golgi, and post-Golgi vesicular transportation is assisted by Rab GTPases, SNARE complexes, and tethering factors, like AP proteins (adaptor proteins), Sec1p–Golgi to plasma membrane, or Sly1–ER to Golgi, specifying the ultimate fate of the cargo proteins (Idiris et al. 2010a; Hong and Lev 2014). The process of protein sorting to the destination membrane is crucial to the organization and functioning of yeast cells and if impaired, leads to multitude dysfunctions (Fig. 2d, e, f). Individual traffic steps and specificity of vesicular fusion at the destination membrane at the traffic cross-roads are subjected to stringent control by numerous intracellular membrane proteins (Idiris et al. 2010a). Nevertheless, several studies conducted on *Schizosaccharomyces pombe* (Idiris et al. 2010b), *S. cerevisiae* (Kitagawa et al. 2011; Wang et al. 2013), *Hansenula polymorpha* (Agaphonov et al. 2005), or *Pichia pastoris* (*Komagataella* spp) (Marsalek et al. 2017) demonstrate significant contribution of mis-sorting events to the overall secretory efficiency of the yeast cell, which could be overcome to some extent by specific genetic manipulations presented in those works. In reference to the scope of this review, occurrence of mis-sorting events has not been studied in *Y. lipolytica*, so far. It would be of great interest to see if *Y. lipolytica* strains impaired in vacuolar sorting could be used for more efficient production of heterologous proteins, as it was shown for the abovementioned yeast species.

## Peculiarities of *Yarrowia lipolytica* secretory machinery

*Y. lipolytica* is a dimorphic yeast, characterized by several unique metabolic properties, when compared to the other yeasts (Barth and Gaillardin 1997; Nicaud 2012). In the following paragraphs, we review and discuss findings on biology of the secretory pathway in this yeast species, as well as other qualities, possibly contributing to enhanced secretory capacity in comparison to the canonical yeast secretory pathway.

First of all, it is thought that dimorphic nature of this species, expressed as the ability to grow in either yeast-like or filamentous forms, is of high relevance for enhanced capacity for protein secretion. This general trait, founded by multitude of specific molecular identities, goes along with a high propensity towards remodeling of cellular membranes and cell wall structures, as well as potent vesicular transportation, altogether directly influencing secretory capacity of this species. In spite of detailed studies on dimorphism-involved genes and phenomena driving dimorphic transition (Szabo and Štofaníková 2002; Morin et al. 2007; Morales-Vargas et al. 2012), no studies adopting this knowledge to engineer *Y. lipolytica* in order to enhance heterologous protein secretion have been published, to date.

In terms of organization of 18S rRNA genes as well as the secretory pathway, *Y. lipolytica* appears to be more closely related to the filamentous fungi than to the other yeast genera (Dujon et al. 2004; Swennen and Beckerich 2007; Swennen et al. 2010). It has been clearly demonstrated by comparative genomics and proteomics approaches that the secretory machinery in *Y. lipolytica* is characterized by significantly higher complexity than the one operating in a typical yeast cell (Swennen and Beckerich 2007; Swennen et al. 2010; Delic et al. 2013). Precisely, the predicted secretome size is twofold higher in *Y. lipolytica* than in the model yeast, *S. cerevisiae* (156 vs 299; number of proteins predicted to have a secretion signal peptide with a subcellular localization predicted as extracellular, but not having a transmembrane domain or an ER targeting signal; curated manually by the Authors) (Delic et al. 2013). Moreover, as revealed by (Swennen and Beckerich 2007), due to several characteristics, like higher representation of Rab GTPase protein families, which is typical for filamentous fungi, multiplied genes encoding membrane ubiquitin ligases that tag the proteins for degradation via ERAD, or increased number of the plasma membrane SNARE complexes (Sso proteins), *Y. lipolytica* is thought to represent a more advanced type of secretory pathway than the non-dimorphic yeast cells. Upon quantitative comparison of sequence similarity of the proteins involved in secretory pathway between *S. cerevisiae*, *Y. lipolytica*, and mammalian homologs, *Y. lipolytica*’s secretory machinery was in 40% more similar to mammalian than to budding yeast’s counterpart (Swennen and Beckerich 2007). Such molecular qualities are considered to underlie the extraordinary capacity of *Y. lipolytica* for production of secretory proteins, which has been long exploited in industrial scale production of heterologous proteins (e.g., prochymosin; European Patent Office application 0220864, European Patent Bulletin 1987/19) (Davidow et al. 1987).

Starting from the beginning of the nascent polypeptide targeting to the secretory pathway, the first fundamental difference between *S. cerevisiae*’s and *Y. lipolytica*’s secretory pathways is visible. As demonstrated by detailed studies, the latter system shows high preference towards co-translational translocation (SRP-dependent) of the nascent polypeptide to the ER lumen, as approximately 75% (vs 30% in *S. cerevisiae*) of the translocation pores (marked by Sec61p) were co-fractionated with ribosomes and Sls1p-Kar2 complexes (ER resident proteins) (Boisramé et al. 1998). In *S. cerevisiae*, an SRP-independent translocation pathway is dominant and essential (Hann and Walter 1991; Rapoport et al. 1999). Moreover, the components of the SRP-dependent targeting were shown to be not essential for *S. cerevisiae* cell survival, as lack of any elements of SRP ribonucleoprotein (either of the six proteins or Scr1 gene encoding 7SL RNA) resulted in slower growth and impairment in protein translocation (as in Fig. 2b), but the cells remained viable (Stirling and Hewitt 1992). In contrast, in *Y. lipolytica*, disruption of the genes encoding the RNA element of SRP is lethal for the cells, demonstrating the crucial role of the SRP-dependent translocation pathway (He et al. 1992). It was discussed that SRP may condition *Y. lipolytica* cell survival, as one or more of the essential proteins involved in the secretory pathway uses co-translational translocation pathway solely, and is not able to follow post-translational translocation route as an alternative (Delic et al. 2013). Noteworthy, *Y. lipolytica* has two functional genes encoding 7SL RNA component of SRP—Scr1 and Scr2 (He et al. 1992; Yaver et al. 1992)—which is typical for higher eukaryotes. On the other hand, only a single gene for Scr1 was identified in *S. cerevisiae*, which also corroborates the statement about higher complexity of *Y. lipolytica*’s secretory pathway. Complementary function and functionality of both Scr1 and Scr2 genes were proved upon individual deletion of either gene, which had no obvious effect on growth or secretion, but double deletions were lethal (He et al. 1992). Moreover, SRP 7SL RNA from *Y. lipolytica* demonstrates higher eukaryotic-like structure than the *S. cerevisiae* homolog in terms of size (270 nt, 300 nt vs 519 nt for *Y. lipolytica, H. sapiens*, and *S. cerevisiae*, respectively) and predicted secondary structure (Sánchez et al. 1997). Furthermore, as studied by Sánchez et al. (1997), deletion of Sec65p, encoding a 19 kDa protein of the SRP complex, is lethal in *Y. lipolytica*, in contrast to deletion of its homolog in *S. cerevisiae*, which results in slowly growing strains, defective in the processing of pre-secretory proteins. Indeed, disruption of any of the SRP components and SRP receptor subunits was shown to be not essential in *S. cerevisiae*, resulting in similar growth reduction, demonstrating secondary importance of this translocation pathway (Hann and Walter 1991; Ogg et al. 1992; Hann et al. 1992; Stirling and Hewitt 1992; Brown et al. 1994; Miller et al. 1995). In contrast to Sec65p, another SRP protein, Srp54, as well as Srp101, an SRP receptor α-subunit (SRαp), was shown to be important but not essential for *Y. lipolytica* growth (Lee and Ogrydziak 1997; Kim and Ogrydziak 2000).

According to genomic sequence analysis conducted by (Delic et al. 2013), *Y. lipolytica* shares similarity with *S. cerevisiae* by bearing two translocon pores, Sec61 and Ssh1. However, it was shown that apart from shared Sec61γ (Sss1p) subunit, *Y. lipolytica* also has a single gene for Sec61β subunit, namely Sbh1/2, which is also shared between the two translocones. As mentioned earlier in this review, Sss1p is the sole common element for both translocation pores in *S. cerevisiae*. Furthermore, with respect to Ssh1-encoding gene from *Y. lipolytica*, it was shown that based on sequence similarity, it is more alike Sec61p rather than members of Ssh1 cluster from the other yeast species studied there (Delic et al. 2013). It is, however, unclear at this moment, how these dissimilarities could influence secretory capacity of *Y. lipolytica* cells. As in the case of *S. cerevisiae*, Kar2 and Sec63 proteins are known to initiate “pulling” the nascent polypeptide in the ER, assist initial folding and gating the pore in *Y. lipolytica* (Kabani et al. 2000). In *S. cerevisiae*, interaction of these two crucial proteins is mediated by NEF proteins, Lhs1 and Sil1, with the leading role attributed to the former, while the role of the latter remained unclear (Steel et al. 2004). In contrast, in *Y. lipolytica*, a homolog of the latter NEF (Sls1) was shown to be directly involved in co-translational translocation (Boisramé et al. 1996, 1998; Kabani et al. 2000). Its deletion *Δsil1*, while having no effect on translocation in *S. cerevisiae*, had strong impact on translocation of nascent secretory proteins in *Y. lipolytica* (depicted as Fig. 2b) (Boisramé et al. 1998). Due to known interaction with another partner protein, Ire1, Sls1 is thought to be involved in sensing and activating unfolded protein response UPR (Boisramé et al. 1996, 1998; Kabani et al. 2000; Babour et al. 2008). These data imply that in *Y. lipolytica*, Sls1 is the dominant NEF, in contrast to a minor role of its homolog Sil1 in *S. cerevisiae*.

*Y. lipolytica*’s secretory machinery exhibits several further dissimilarities when compared to the model yeast’s system with respect to ER-resident proteins. One of the key features concerns protein disulfide isomerase family, PDIs. As reported earlier (Delic et al. 2013), PDI family is represented by five members in *S. cerevisiae*, out of which, only three share homology with *Y. lipolytica* counterparts (essential Pdi1, Mdp1, and Eps1). Strikingly, genome of *Y. lipolytica* codes for a PDI family member, ERp38, which is well conserved in filamentous fungi and some hemiascomycete yeast, but is missing in *S. cerevisiae*’s and its closely related species. While still not much is known about its specific functions, in *Neurospora crassa*, ERp38 was shown to interact with Pdi1 and with Kar2 (Tremmel et al. 2007). There is increasing evidence that chaperones and folding catalysts in the ER act together by forming complexes to fold nascent proteins (Tremmel et al. 2007). Based on such an interaction between Mdp1 (having oxidative folding activity) and calnexin (Cne1; involved in glycosylation) (Kimura et al. 2005), the former was postulated to play a role in disulfide bond formation of glycoproteins.

Another distinguishing trait of *Y. lipolytica*’s secretory pathway is the presence of an operable calnexin cycle in the ER lumen which was not identified in *S. cerevisiae* (Boisrame et al. 2002; Babour et al. 2004; Swennen and Beckerich 2007; Swennen et al. 2010; Delic et al. 2013) (compare Fig. 1). In fact, the gene encoding calnexin (Cne1) is present in *S. cerevisiae* genome. However, for full operation, the cycle requires UDP-glucose:glycoprotein glucosyltransferase (UGGT), and this activity is not encoded in its genome (Babour et al. 2004). UGGT acts as a folding sensor and enables re-binding of calnexin to the partially misfolded polypeptide, which extends the time of a given protein residence in the ER to fold correctly (Delic et al. 2013). Thus, the calnexin cycle is thought to serve as a decision branch point between ERAD, longer residence in the ER, or further transportation of glycosylated proteins. Glycosylation of proteins secreted outside the cell or associated with cell wall or plasma membrane endows them with novel traits, like increased solubility and stability, which further affects stability of cell wall, osmotolerance, and budding (Delic et al. 2013). The calnexin (Cnx1p) itself is an ER-resident, membrane-bound chaperone displaying a large N-terminal lumenal domain, playing a role in folding of glycoproteins. In *Y. lipolytica*, Cnx1p is well conserved when compared to other calnexin sequences and displays 45% identity to human calnexin (Boisrame et al. 2002). By screening two-hybrid library for interaction partners, the lumenal domain of calnexin was shown to interact with a Sec61β (Sbh1) translocon subunit (Boisrame et al. 2002). It was stated that in *Y. lipolytica*, and in the other systems bearing the calnexin chaperone, the role of Sec61β in the quality control of secretory proteins could consist of maintaining the chaperone calnexin in the vicinity of the translocation pore, and by this, linking the translocon with folding and/or quality control of secretory proteins. Such a proximity allows calnexin to interact with some nascent polypeptide chains as soon as they emerge in the ER lumen. Deletion of *ΔSec61β* in *Y. lipolytica* (the absence of the docking protein) would therefore lead to uncoupling of translocation and quality control, and misfolded polypeptides would no longer be retained in the ER compartment; nevertheless, no secretory defects were observed experimentally in *ΔSec61β* genotype background (Boisrame et al. 2002), possibly suggesting existence of complementary mechanisms. As extensively discussed in De Pourcq et al. (2012) and Delic et al. (2013), the initial ER-resident glycosylation functions (OST and Pmt complexes) are rather conserved, while the following glycosylation events in the Golgi compartment are highly variable between different yeasts, and thus, it seems difficult to identify specific qualities of a single species. However, *Y. lipolytica* is frequently claimed to bear the glycosylation pattern closer to human than *S. cerevisiae* (Madzak et al. 2004; Nicaud 2012). Indeed, as reported by Delic et al. (2013), the genomic sequence of *Y. lipolytica* lacks MNN1 (α-1,3 mannosyltransferase) family representatives and bears only limited number of KTRs (α-1,2 mannosyltransferase), whose activity leads to overmannosylation of secreted polypeptides in yeast (De Pourcq et al. 2012).

Correctly folded proteins are concentrated at transitional ER sites (ERES) where COPII elements assemble. Prior to being packed into COPII vesicle, secretory proteins are recognized by either COPII pre-budding complex subunits or specific cargo receptors and anchors. The initial steps of routing cargo proteins from ER are well conserved, with minor differences observed for *Y. lipolytica*, like lack of Yos1, a Rab-type GTPase, or Cog1 and Cog7, tethering complexes (Delic et al. 2013). No specific details on structural organization of ER and Golgi compartment are known for *Y. lipolytica*. It would be interesting to see whether it exhibits greater similarity to *S. cerevisiae* (dispersed tER sites and scattered Golgi cisternae) or *Pichia pastoris* (discrete number of tER sites and Golgi stacks) (Delic et al. 2013) in this regard.

Unique qualities of the *Y. lipolytica*’s protein managing system were identified also amongst events occurring in the cytosol, expressed for example in specificities of cytosolic chaperones. Cytosolic Hsp70s and their Hsp40s co-chaperones play essential roles in protein folding, transportation, or degradation, and constitute major players of cellular quality control processes. Based on genomic sequence analysis, it was stated that *S. cerevisiae* bear doubled set of Sse and Ssb chaperones—one stress-induced and the other constitutive isoform, which is not the case in *Y. lipolytica* (Delic et al. 2013). The number of Ssa cytosolic chaperones was the same for both species. Nevertheless, systematic, comparative studies on eight cytosolic chaperones, Ssa, from *S. cerevisiae* (Ssa1-4p) and *Y. lipolytica* (Ssa5-8p) demonstrated that despite a high degree of sequence homology, individual representatives of Ssa family possess redundant yet clearly distinct functional properties, and some degree of species-specificity (Sharma et al. 2009). Noteworthy, even though sequence similarity was more pronounced amongst the four Ssa orthologs from *Y. lipolytica*, the ability to support growth in deletion/overexpression strains was more variable between different variants than in *S. cerevisiae*, indicating higher functional specialization of Ssas in this species (Sharma et al. 2009). To further stress the uniqueness of *Y. lipolytica*’s secretory machinery spanning also cytosolic events, it is worth mentioning that *Y. lipolytica* is the only so far identified hemiascomycete bearing of a Hsc70-interacting protein (CHIP) (Kabani and Martineau 2008). CHIP orthologs are found in many filamentous fungi, but they are generally absent from yeast systems. CHIP is a multifunctional protein playing an important role in quality control of protein folding by providing a link between the protein folding pathway and the UPR (unfolded protein response). It was also demonstrated that Yl.Chn1p is non-essential unless *Y. lipolytica* is severely stressed (Martineau et al. 2012) indicating operation of complementary pathways securing survival of the cells. It was demonstrated that Yl.Chn1p interacts with Ssa1p (cytosolic Hsp70 molecular chaperone), and this interaction is Fes1p-dependent (a nucleotide exchange factor of Ssa1p). In terms of Yl.Chn1p function, it was concluded that it can act as a “holdase” to prevent the aggregation of a heat-denatured proteins (Martineau et al. 2012).

## Engineering secretory capacity of *Yarrowia lipolytica*

Although in many studies, *Y. lipolytica* has been proved to be a competitive protein secretor when compared to the conventional yeast host—*S. cerevisiae* (Barth and Gaillardin 1996; Steinborn et al. 2005; Ogrydziak and Nicaud 2012; Madzak 2018), some individual studies demonstrate that this field still leaves some potential for improvement. Enhanced production of the targeted, secretory protein may be achieved by several different strategies, including (i) engineering the DNA coding sequence itself, (ii) modification of a genetic construct structure, (iii) optimization of culturing conditions, and (iv) engineering molecular mechanisms of protein synthesis and secretion pathway (Graf et al. 2009; Liu et al. 2012). Within each of the proposed strategies, various solutions have been proposed, and some of them were conducted in *Y. lipolytica*, like (i) optimization of codon usage within the coding sequence (Celińska et al. 2015; Celińska 2015; Dulermo et al. 2017), (ii) amplification of the gene copy number, manipulation with regulatory elements contained in the genetic construction, mainly promoter (Le Dall et al. 1994; Gasmi et al. 2011b; Gasmi et al. 2011a; Gasmi et al. 2012; Dulermo et al. 2017; Larroude et al. 2018), (iii) bioprocess engineering, optimization of cultivation conditions (Chang et al. 1998; Kim et al. 2000; Nicaud et al. 2002; Celińska et al. 2017a), and (iv) co-synthesis of chaperones, manipulation with protein folding, and maturation mechanisms, engineering secretory tags (De Pourcq et al. 2012; Celińska et al. 2018). Nevertheless, the vast majority of studies on enhanced production of secretory proteins in *Y. lipolytica* was focused on strategies from (i) to (iii), while the approaches directing secretory pathway were largely neglected. On the other hand, the amount of knowledge concerning molecular details of the secretory pathway in this species seems to be sufficient to facilitate further engineering strategies. The key reports are summarized and discussed below.

Considerable effort was devoted to elucidation of mechanisms driving translocation, maturation, and secretion of one of the two major secretory proteins in *Y. lipolytica* secretome—alkaline extracellular protease (AEP, encoded by XPR2 gene), which became a model protein within this research area (Matoba et al. 1988; Matoba and Ogrydziak 1989; Fabre et al. 1991; Fabre et al. 1992; He et al. 1992; Yaver et al. 1992; Le Dall et al. 1994; Matoba et al. 1997; Matoba and Ogrydziak 1998; Ogrydziak and Nicaud 2012). As such, most of initial modifications of *Y. lipolytica* secretory pathway were realized using this model protein. It was for example revealed, that AEP follows co-translational translocation pathway and is efficiently translocated and rapidly (several minutes) excreted outside the cell (Matoba et al. 1988; Matoba and Ogrydziak 1989; Yaver et al. 1992); AEP undergoes maturation via three consecutive intracellular precursors—the products of catalytic activity of signal peptidase and oligosaccharyl transferase in the ER (55 kDa) (Matoba et al. 1988), dipeptidyl aminopeptidase in the late Golgi (52 kDa) (Matoba and Ogrydziak 1989), and Kex2-like protease XPR6 in the late Golgi as well (32 kDa) (Matoba et al. 1988). Those and also the other studies (Fabre et al. 1991; Fabre et al. 1992; Yaver et al. 1992) lead to the elucidation and understanding of the crucial N-terminal domain structure and function of its individual elements. It was demonstrated that the AEP protein is initially synthesized as prepro-polypeptide, and the identified intracellular precursors are intermediates devoid of either pre- or pro-domains. The architecture of the prepro-domain in AEP corresponds to the typical structure of a yeast leader sequence (Yang et al. 2006; Yarimizu et al. 2015), covering an N-domain bearing at least one positively charged amino acid residue, followed by a hydrophobic H-domain composed of a tract hydrophobic amino acid residues forming an alpha-helix, which is essential for translocation of the polypeptide through the membrane, terminated with a C-domain containing an alpha-helix-breaking or polar residue, facilitating digestion through a specific signal peptidase, ended with a consensus sequence AX-A (X-any residue), which is recognized by the ER-localized signal peptidase. Detailed studies on pre- and pro-domains separately allowed to dissect their specific functions in processing of AEP in the secretory pathway and founded a background to be applied in engineering strategies aiming at enhanced production of secretory proteins.

The pre-sequence is an interaction partner of SRP and hence is responsible for directing the polypeptide for translocation into the ER lumen. It was elegantly demonstrated that specific properties of the pre-domain governed by its primary structure significantly influence the rate of secretion and can specify the translocation pathway followed by the nascent polypeptide (Yaver et al. 1992; Matoba and Ogrydziak 1998). It was inferred that conformation/orientation of the pre-domain and its hydrophobicity are the key parameters affecting its interaction with SRP and consequently the designated pathway for translocation in *Y. lipolytica* (Matoba and Ogrydziak 1998). It was shown that P17M mutation (loss of kinked secondary structure) disallows pre-domain-SRP interaction and leads to post-translational translocation of the AEP, which was kinetically less efficient, but nevertheless allowed secretion of the protein via its “non-native” translocation pathway (Yaver et al. 1992). While such data are not available for *Y. lipolytica*, it was calculated that in *S. cerevisiae*, more hydrophobic pre-domains (HB12 value ≥ 3.0) are preferentially translocated via the SRP-dependent pathway, while less hydrophobic leaders (HB12 ≤ 2.0) are processed via the post-translational translocation pathway (Ng et al. 1996). However, as demonstrated in *Y. lipolytica*, based on analyses of a panel of mutated pre-domains of AEP comprising leaders differing in hydrophobicity and secondary structure, it was inferred that a model involving only average hydrophobicity and kinkiness does not accurately explain the observed variability in the experimental data (Matoba and Ogrydziak 1998), indicating influence of some other, unknown factors.

The pro-domain in AEP’s N-terminus, bearing N-glycosylation sites, assists folding and maturation of the polypeptide, before it is packed in the vesicles for exocytosis. It was demonstrated that deletion of the glycosylation site within the pro-domain yields unglycosylated AEP precursors which were matured and secreted correctly at 18 °C but remained trapped in the secretory pathway as unprocessed forms at 28 °C (Fabre et al. 1991). The trapped precursors could not be processed by the dipeptidyl aminopeptidase and the Kex2-like XPR6 endoprotease and thus were not secreted (Fabre et al. 1991). Interestingly, secretion of such AEP polypeptides (modified in the pro-domain) could be rescued when the pro-domain was supplied in trans as an independent peptide (Fabre et al. 1992). From that study, it was inferred that the pro-domain acts as molecular chaperone by direct interaction with the mature region of AEP and participates in acquisition of a catalytically active conformation until late into the secretory pathway (Fabre et al. 1992). Nevertheless, it was proved that the pro-domain of AEP is not obligatory needed for secretion of heterologous proteins, and in some cases may be even deleterious for such a process (Nicaud et al. 1989; Fabre et al. 1991; Park et al. 1997, 2000; Boisramé and Gaillardin 2009; Hong et al. 2012).

All those detailed studies founded a background for rational design of production processes for heterologous secretory proteins in *Y. lipolytica*. Up to date, most of heterologous secretory proteins have been directed to the secretory pathway via AEP-derived or LIP2-derived pre-sequences (the two major secretory proteins), or their corresponding hybrids (Madzak and Beckerich 2013; Madzak 2015; Madzak 2018). As mentioned earlier, a sole AEP pre-leader was proved to efficiently target the polypeptide to the secretory pathway, without the need for a pro-leader. Native Lip2 prepro domains (Pignede et al. 2000), its hybrid with AEP pre-sequence (Nicaud et al. 2002), or synthetic leader sequences issued from the LIP2 pre-domain (Gasmi et al. 2011c; Gasmi et al. 2012; Ledesma-Amaro et al. 2015) were all proved to operate with high efficiency in *Y. lipolytica*’s secretory pathway with different heterologous polypeptides. In several studies, it was reported that heterologous pre-domains can efficiently operate in *Y. lipolytica* secretory pathway, including fungal and yeast leaders (Müller et al. 1998; Hong et al. 2012), plant secretory elements (Park et al. 1997), or even such domains originating from phylogenetically distant insects (Celińska et al. 2015, 2016a). In our efforts towards optimization of heterologous production of insect-derived, raw starch digesting alpha-amylase (SoAMY; *Sitophilus oryzae* (Celińska et al. 2016b)) in *Y. lipolytica* system, we tested different N-terminal fusions with secretory tags, comprising pre-AEP, pre-LIP2, native pre-domain of SoAMY, as well as hybrid fusion pre-AEP-native pre-domain (Celińska et al. 2016a, 2017b). We observed high efficiency of secretion upon transcriptional fusion of the mature SoAMY polypeptide with pre-AEP, and unsuitability of native pre-LIP2 in this regard. In our recent study (Celińska et al. 2018), we analyzed the potential of ten different pre-domains towards targeting of two model polypeptides to the secretory pathway. The pre-sequences under study covered those well-known, like pre-LIP2 or pre-AEP, some previously described, like the hybrid pre-LIP2 or the insect-derived pre-SoAMY, but most importantly novel pre-sequences, previously undescribed in the context of recombinant protein secretion in *Y. lipolytica*. The novel secretory tags were identified through genomic DNA data mining and hence constituted homologous elements for *Y. lipolytica* secretome, comprising YALI0B03564p (putative product: 1,3-beta-glucosidase precursor), YALI0D20680p (cell wall protein with similarity to glucanases), YALI0E22374p (YPS3, GPI-anchored aspartyl protease 3), YALI0D06039p (PHR1, alkaline cell surface glycosidase), and YALI0D06149p (sterol binding protein involved in the export of acetylated sterols). Based on the obtained experimental data, it could be inferred that four out of five novel secretory tags compare favorably in terms of targeting of both reporter proteins to the secretory pathway, when compared to the conventional elements pre-LIP2 and pre-AEP. Since the secretory elements under study worked with corresponding efficiency for both proteins, it was concluded that the observed results may potentially reflect general potency of the pre-domains itself in driving secretion of polypeptides in *Y. lipolytica* systems and that the analyzed pre-domains, and not the following mature polypeptide, had the dominant influence on the observed variation in efficiency of the proteins secretion. Although in a single case, we could clearly see dependence of the type of the following reporter protein on such efficiency (heterologous pre-domain from fungi—*Thermomyces lanuginosus*); such observation has been previously made upon *S. cerevisiae*’s Suc2 invertase production in *Y. lipolytica* (Hong et al. 2012). Since it was earlier reported that hydrophobicity and secondary structure are the key factors determining efficiency of the pre-sequence in driving the nascent polypeptide translocation and specifying the translocation mode (Yaver et al. 1992; Matoba and Ogrydziak 1998), we used a panel of computational tools to study these parameters in the analyzed secretory elements. However, we could not see any straightforward correlation between the average hydrophobicity and the final extracellular abundance of the reporters (Celińska et al. 2018). Correspondingly, neither calculated D-score values (reflecting probability of being recognized and processed by signal peptidase) nor predicted secondary structure could provide the key or support the rules governing the observed results. Similar conclusions were inferred from results on AEP secretion equipped with mutated versions of its own pre-domain (Matoba and Ogrydziak 1998).

## Trends and future prospects

*Y. lipolytica* has already gained established position as an industrial workhorse and highly efficient platform for heterologous protein production (Madzak 2018). Nevertheless, many strategies of improving secretory capacity that were executed for the other yeast species, like *S. cerevisiae* (Idiris et al. 2010a) or *P. pastoris* (Zahrl et al. 2017), remained largely neglected in *Y. lipolytica*. Such a situation leaves a wide array of approaches to be tested in this system. Having the knowledge and background provided by the aforementioned detailed studies, this field is now open to enter with new strategies. For this moment, it is hard to foresee, which strategy may turn out to be superior for *Y. lipolytica*. Manipulation with vesicular traffic (Swennen and Beckerich 2007; Idiris et al. 2010b; Puxbaum et al. 2015; Marsalek et al. 2017) or relief of stress imposed by production of heterologous protein (Mattanovich et al. 2004; Gasser et al. 2007a) seems to be particularly attractive approaches. It might be also useful to refer to the corresponding research conducted with filamentous fungi, like (Ohno et al. 2011; Liu et al. 2014), due to high similarities in the secretory pathways of *Y. lipolytica* and filamentous fungi, as elaborated on in this review.
